# Association Between Specific Type 2 Diabetes Therapies and Risk of Alzheimer’s Disease and Related Dementias in Propensity-Score Matched Type 2 Diabetic Patients

**DOI:** 10.3389/fnagi.2022.878304

**Published:** 2022-05-06

**Authors:** Georgina Torrandell-Haro, Gregory L. Branigan, Roberta Diaz Brinton, Kathleen E. Rodgers

**Affiliations:** ^1^Center for Innovation in Brain Science, University of Arizona, Tucson, AZ, United States; ^2^Department of Pharmacology, University of Arizona College of Medicine, Tucson, AZ, United States; ^3^MD-PhD Training Program, University of Arizona College of Medicine, Tucson, AZ, United States; ^4^Department of Neurology, University of Arizona College of Medicine, Tucson, AZ, United States

**Keywords:** type 2 diabetes, Alzheimer’s disease, vascular dementia, anti-hyperglycemic agents, risk analyses

## Abstract

**Objective:**

We sought to determine the impact of Type 2 Diabetes Mellitus (T2D) anti-hyperglycemic medications (A-HgM) on risk of Alzheimer’s disease (AD) and related dementias (ADRD) outcomes including vascular dementia, and non-AD dementia such as frontotemporal, Lewy body, and mixed etiology dementias.

**Research Design and Methods:**

This retrospective cohort study used the US-based Mariner claims dataset. 1,815,032 T2D participants 45 years and older with records 6 months prior and at least 3 years after the diagnosis of T2D were included. Claims were surveyed for a diagnosis of AD and ADRD 12 months post T2D diagnosis. A propensity score approach was used to minimize selection bias. Analyses were conducted between January 1st and February 28th, 2021.

**Results:**

In this cohort study A-HgM exposure was associated with decreased diagnosis of AD (RR, 0.61; 95% CI, 0.59–0.62; *p* < 0.001), vascular dementia (RR, 0.72; 95% CI, 0.69–0.74; *p* < 0.001) and non-AD dementia (RR, 0.67; 95% CI, 0.66–0.68; *p* < 0.001). Metformin was associated with the greatest risk reduction and insulin with the least reduction in risk compared to patients not receiving A-HgM for ADRD risk. Of interest, patients with a diagnosis of AD, while either on metformin or insulin, were older in age and predominately female, than individuals on these drugs that did not develop AD. Mean (SD) follow-up was 6.2 (1.8) years.

**Conclusion:**

After controlling for age, sex, and comorbidities, A-HgM in patients with T2D was associated with a reduced risk of AD and ADRD. These findings provide evidence in support of T2D as a risk factor for AD and ADRD and the beneficial impact of early and effective control of hyperglycemia to mitigate risk.

## Introduction

Type 2 diabetes (T2D) is a lifestyle-associated disease with widespread prevalence (International Diabetes Federation, 2019). The hallmark of T2D is dysregulation of glucose metabolism resulting in hyperglycemia. According to a recent report by International Diabetes Federation (2019), the number of patients with T2D worldwide was 463 million in 2019, indicating that one in eleven adults are diabetic. However, this statistic is likely an underestimate of the burden of this disease as it is predicted that, worldwide, one in two patients with diabetes are undiagnosed in most under-developed and developing countries (Cho et al., 2018).

Multiple population-based studies have reported an association between T2D and cognitive impairment (Hassing et al., 2004; Strachan, 2011), Alzheimer’s disease (AD) (Li et al., 2015; Sun et al., 2020) and vascular dementia (VasD) (Cholerton et al., 2016; Teixeira et al., 2020). Factors that contribute to this increased risk include cardiovascular disease, hyperglycemia, inflammation, and oxidative stress (Rosales-Corral et al., 2015; Willette et al., 2015; Ferreira et al., 2018). Further, middle-aged and older adults with T2D experience a more rapid cognitive decline than those without T2D (McCrimmon et al., 2012).

Dementia, AD and VasD are progressive neurodegenerative disorders that manifest in impaired cognition, executive function, psychiatric symptoms and loss of independent living (2020). Dementia affects approximately 50 million people globally and is rapidly increasing in prevalence with AD and VasD as the most prevalent forms of dementia (2020). Late-onset AD (LOAD) primarily affects persons 65 years of age and older (2020). Risk factors of LOAD include APOE4 genotype, mid-life hypertension, mid-life metabolic dysregulation (metabolic syndrome and T2D), stroke and brain trauma (Edwards III et al., 2019). VasD is primarily caused by impaired cerebral blood flow or cerebrovascular disease, including stroke (Smith, 2017).

Medications that restore glucose control in T2D act through multiple mechanisms (Inzucchi et al., 2012). Broadly, anti-hyperglycemic medications (A-HgM) are divided into four main categories based on mechanism of action and include: (1) insulin sensitizers (biguanides and glitazones), (2) insulin secretagogues (sulfonylureas and meglitinides), (3) incretin mimetics (GLP1 agonists and DPP4 inhibitors) and (4) insulin. Insulin is indicated to patients who fail to meet glycemic targets on multiple non-insulin therapeutics, patients with catabolic symptoms suggestive of insulin deficiency or patients with advanced disease who have lost significant endogenous insulin secretory capacity.

Multiple studies have evaluated the effect of metformin on cognitive decline with mixed results (Moore et al., 2013; Herath et al., 2016; Orkaby et al., 2017; Scherrer et al., 2019; Samaras et al., 2020). Metformin, which is first line therapy for the treatment of T2D, is a biguanide in the insulin sensitizer class (Nasri and Rafieian-Kopaei, 2014). Metformin concentrates in the mitochondria due to its positive charge (Nasri and Rafieian-Kopaei, 2014). Mitochondrial dysfunction is well documented as a potential phenotype during the prodromal phase of AD making metformin an interesting target in AD treatment (Chakravorty et al., 2019; Wang et al., 2020). Studies have shown a benefit of metformin, particularly in comparison to the sulfonylurea medication class in reducing the risk of AD such that many studies use metformin as the control group for comparison with other A-HgM agents (Rizzo et al., 2014; Orkaby et al., 2017). In a recent study involving approximately 2,000 patients with T2D, metformin use was associated with better memory performance over time and dipeptidyl peptidase-4 (DDP-4) inhibitors slowed the rate of memory decline with the greatest benefit in APOE4 carriers (Wu et al., 2020).

This study sought to determine the impact of A-HgM overall and as individual mechanistic drug classes on the incidence of newly diagnosed AD, VasD or non-AD dementia outcomes in patients with T2D over the age 45. The comparator group used for this study was patients with T2D without exposure to A-HgM agents and control their diabetes with lifestyle modifications. Analyses were conducted using a US-based population insurance claims dataset and a substantially larger number of patients than previously reported (Moore et al., 2013; Orkaby et al., 2017; Samaras et al., 2020). The majority of studies involved small number of patients (from 80 to 1455). The two larger studies involving tens of thousands of patients involved the evaluation of a largely male population (veterans) (Orkaby et al., 2017; Scherrer et al., 2019). Herein, we report the association of individual A-HgM agents and their drug class families with reduced risk of development of age-associated AD, VasD and Non-AD dementia. Further, we conducted a responder’s analysis to identify and describe characteristics of patient groups who exhibited benefit vs. no benefit for AD outcomes with respect to A-HgM exposure.

## Research Design and Methods

### Data Source

The Mariner database is an insurance claims dataset that serves the United States with patient populations from all U.S. states and territories. Pearl Diver is for-fee research software that facilitates interaction with individual commercial, state-based Medicaid, Medicare stand-alone prescription drug plan, group Medicare Advantage, and individual Medicare Advantage data. The Mariner dataset contains patient demographic characteristics, prescription records, and numerous other data points for patients with *Current Procedural Terminology*, *International Classification of Diseases, Ninth Revision (ICD-9)*, and *International Statistical Classification of Diseases and Related Health Problems, Tenth Revision (ICD-10)* codes. As of October 2020, Mariner encompasses all indications and represents 122 million patients throughout the duration of the set with claims from 2010 through the second quarter of 2018.

This report follows the Strengthening the Reporting of Observational Studies in Epidemiology (STROBE) reporting guideline. This study was approved by the University of Arizona Institutional Review Board. Requirements for informed consent were waived as the data were deidentified.

### Study Design and Variables

**FIGURE 1:**
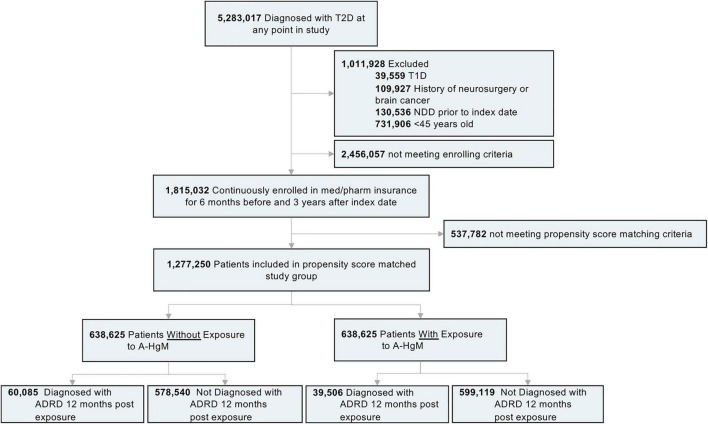
Study design and patient breakdown. T2D, type 2 diabetes; T1D, type 1 diabetes; NDD, neurodegenerative diseases; A-HgM, anti-hyperglycemic medication; ADRD, Alzheimer’s disease related dementias.

The subset of 5,283,017 patients with Type 2 Diabetes Mellitus (T2D) were selected from the available Mariner dataset based on the presence of greater than one ICD code for T2D (Table 1). The outcome variable was defined as the occurrence of the first diagnosis of AD, VasD, or non-AD dementia based on ICD-9 and ICD-10 codes in the patient’s medical claims data (Table 2). An index date one year after the diagnosis of T2D was selected to allow for onboarding to therapy and to focus on long-term impact on disease progression given the prodromal nature of these neurodegenerative diseases. Participants younger than 45 years old, with a diagnosis of Type 1 Diabetes, or with a history of neurosurgery, brain cancer, or neurodegenerative disease prior to the index date were excluded from the study. An enrollment criterion in the claims dataset of at least 6 months prior to and 3 years after the diagnosis of T2D was applied. The enrollment criteria was required before any analysis of exposure to A-HgM for development of AD and ADRD for all patients to account for patients that may be leaving, dying, or switching the insurance provider (Figure 1). Additionally, the 3-year follow-up is not with respect to A-HgM exposure but instead with respect to T2D diagnosis. Patient groups were defined according to the therapeutic intervention used. The treatment group is defined as patients having at least one medication charge for an anti-hyperglycemic agent occurring after the diagnosis of T2D. This group was then stratified based on the mechanism of action of each therapeutic intervention for T2D (Tables 1, 3). The A-HgM group was defined as patients with one or more codes for insulin, metformin, glitazones, sulfonylureas, sodium-glucose cotransporter-2 (SGLT2) inhibitors, glucagon-like peptide (GLP-1) agonists, DPP4 inhibitors, glinides, or combination therapies (e.g., metformin and sulfonylureas) (Table 3). Drug groups with a low number of patients were not included in the sensitivity analysis evaluating the association between AD and ADRD and individual drug groups but were included in the relative risk analysis of overall A-HgM. Median adherence rates for each drug as well as the duration of treatment are reported in Table 1. Age in the study is defined by the age at diagnosis of T2D. Following the analysis in Branigan et al. (2020) and Torrandell-Haro et al. (2020), an analysis of comorbidities known to be associated with AD and ADRD outcomes was conducted (Table 2) which was used to generate a logistic regression-based propensity score matched cohort for treatment and control populations.

**TABLE 1:** List of anti-hyperglycemic therapeutics and number of patients.

Class	Group	Drug	Median adherence (%)	*n* (%)	Median time ± SD Duration of treatment (years)
Insulin	Insulin	Insulin	47.59%	250,811 (15.33)	7.80 ± 1.80
Insulin Sensitizers	Biguanides	Metformin	65.92%	523,758 (32.01)	7.87 ± 1.80
	Glitazones	Pioglitazone	80.54%	87,021 (5.32)	8.47 ± 1.74
		Rosiglitazone	93.08%		
Insulin secretagogues	Sulfonylureas	Glyburide	76.98%	292,155 (17.86)	7.91 ± 1.76
		Glimepiride	77.14%		
		Glipizide	75.00%		
		Tolazamide	95.34%		
		Tolbutamide	95.74%		
		Chlorpropamide	87.16%		
Incretin mimetics	DPP4 Inhibitors	Alogliptin	76.48%	145,386 (8.89)	7.68 ± 1.71
		Linagliptin	81.97%		
		Saxagliptin	85.27%		
		Sitagliptin	79.47%		
Combination therapies	Metformin and Sulfonylureas	Glipizide/Metformin	78.28%	257,053 (15.71)	NA
		Glyburide/Metformin	73.81%		
	Metformin and Glitazone	Pioglitazone/Metformin	75.58%	79,880 (4.88)	NA
		Rosiglitazone/Metformin	87.21%		

*DPP4, dipeptidyl-peptidase 4.*

**TABLE 2:** List of diagnose codes used.

Diagnosis	ICD-9	ICD-10
Alzheimer’s disease	ICD-9-D-3310	ICD-10-D-G300, ICD-10-D-G301, ICD-10-D-G308, ICD-10-D-G309
Amyotrophic lateral sclerosis (ALS)	ICD-9-D-33520	ICD-10-D-G1221
Asthma	ICD-9-D-49300, ICD-9-D-49399	ICD-10-D-J452:ICD-10-D-J45988
COPD	ICD-9-D-490:ICD-9-D-49699	ICD-10-D-J44:ICD-10-D-J449
Chronic kidney disease	ICD-9-D-585, ICD-9-D-5851, ICD-9-D-5852, ICD-9-D-5853, ICD-9-D-5854, ICD-9-D-5855, ICD-9-D-5856, ICD-9-D-5859, ICD-9-D-7925	ICD-10-D-N18:ICD-10-D-N189
Congestive heart failure	ICD-9-D-39891, ICD-9-D-4280, ICD-9-D-4281, ICD-9-D-42820, ICD-9-D-42821, ICD-9-D-42822, ICD-9-D-42823, ICD-9-D-42830, ICD-9-D-42831, ICD-9-D-42832, ICD-9-D-42833, ICD-9-D-42840, ICD-9-D-42841, ICD-9-D-42842, ICD-9-D-42843, ICD-9-D-4289	ICD-10-D-I150:ICD-10-D-I159
Coronary artery disease	ICD-9-D-4110:ICD-9-D-4149	ICD-10-D-I25:ICD-10-D-I259
Diabetes type 1	ICD-9-D-24901, ICD-9-D-24911, ICD-9-D-24921, ICD-9-D-24931, ICD-9-D-24941, ICD-9-D-24951, ICD-9-D-24961, ICD-9-D-24971, ICD-9-D-24981, ICD-9-D-24991, ICD-9-D-64802, ICD-9-D-64803, ICD-9-D-64804, ICD-9-D-64800, ICD-9-D-64801	ICD-10-D-E1010, ICD-10-D-E1022, ICD-10-D-E1029, ICD-10-D-E10311, ICD-10-D-E10319, ICD-10-D-E10321, ICD-10-D-E103211, ICD-10-D-E103212, ICD-10-D-E103213, ICD-10-D-E103219, ICD-10-D-E10329, ICD-10-D-E103291, ICD-10-D-E103292, ICD-10-D-E103293, ICD-10-D-E103299, ICD-10-D-E10331, ICD-10-D-E103311, ICD-10-D-E103312, ICD-10-D-E103313, ICD-10-D-E103319, ICD-10-D-E10339
Diabetes type 2	ICD-9-D-25000, ICD-9-D-25002, ICD-9-D-25060, ICD-9-D-25001, ICD-9-D-25080, ICD-9-D-25040, ICD-9-D-25050, ICD-9-D-25070, ICD-9-D-25062, ICD-9-D-25003, ICD-9-D-25042, ICD-9-D-25090, ICD-9-D-25082, ICD-9-D-25092, ICD-9-D-25061, ICD-9-D-25052, ICD-9-D-25072, ICD-9-D-25010, ICD-9-D-25051, ICD-9-D-25013, ICD-9-D-25081, ICD-9-D-25012, ICD-9-D-25071, ICD-9-D-25041, ICD-9-D-25063, ICD-9-D-25091, ICD-9-D-25043, ICD-9-D-25011, ICD-9-D-25083	ICD-10-D-E119, ICD-10-D-E1165, ICD-10-D-E1140, ICD-10-D-E1122, ICD-10-D-E1142, ICD-10-D-E1169, ICD-10-D-E118, ICD-10-D-E1151, ICD-10-D-E109, ICD-10-D-E1129, ICD-10-D-E1065, ICD-10-D-E1159, ICD-10-D-E1149, ICD-10-D-E11649, ICD-10-D-E11621, ICD-10-D-E1143, ICD-10-D-E11319, ICD-10-D-E113293, ICD-10-D-E1100, ICD-10-D-E11329, ICD-10-D-E1139, ICD-10-D-E1010, ICD-10-D-E1040, ICD-10-D-E1136, ICD-10-D-E11622, ICD-10-D-E1042, ICD-10-D-E1152, ICD-10-D-E108, ICD-10-D-E1141, ICD-10-D-E1022, ICD-10-D-E11628
Hypertension	ICD-9-D-4010:ICD-9-D-4059	ICD-10-D-I10:ICD-10-D-I159
Ischemic heart disease	ICD-9-D-41000:ICD-9-D-41499	ICD-10-D-I21:ICD-10-D-I229
Multiple sclerosis	ICD-9-D-340	ICD-10-D-G35
Neurosurgery or brain cancer	ICD-9-D-01320, ICD-9-D-01321, ICD-9-D-01322, ICD-9-D-01323, ICD-9-D-01325, ICD-9-D-01326, ICD-9-D-01330, ICD-9-D-01333, ICD-9-D-1917, ICD-9-D-1918, ICD-9-D-1919, ICD-9-D-1983, ICD-9-D-2250, ICD-9-D-2375, ICD-9-D-2396, ICD-9-D-3481, ICD-9-D-3484, ICD-9-D-34882, ICD-9-D-7422, ICD-9-D-V1085, ICD-9-D-V1241	ICD-10-D-A066, ICD-10-D-A1781, ICD-10-D-A5482, ICD-10-D-B431, ICD-10-D-C717, ICD-10-D-C718, ICD-10-D-C719, ICD-10-D-C7931, ICD-10-D-D330, ICD-10-D-D331, ICD-10-D-D332, ICD-10-D-D430, ICD-10-D-D431, ICD-10-D-D432, ICD-10-D-D496, ICD-10-D-G931, ICD-10-D-G935, ICD-10-D-G9382, ICD-10-D-S06317A, ICD-10-D-S06317S, ICD-10-D-S06327A, ICD-10-D-S06337A, ICD-10-D-S06337S, ICD-10-D-S06377A, ICD-10-D-S06380A, ICD-10-D-S06380D, ICD-10-D-S06380S, ICD-10-D-S06381A, ICD-10-D-S06381D, ICD-10-D-S06382S, ICD-10-D-S06384A, ICD-10-D-S06385S, ICD-10-D-S06387A, ICD-10-D-S06387S, ICD-10-D-S06389A, ICD-10-D-S06389D, ICD-10-D-S06389S, ICD-10-D-Z85841, ICD-10-D-Z86011
Non-Alzheimer’s dementia	ICD-9-D-2900, ICD-9-D-29010, ICD-9-D-29011, ICD-9-D-29012, ICD-9-D-29013, ICD-9-D-29020, ICD-9-D-29021, ICD-9-D-2903, ICD-9-D-29040, ICD-9-D-29041, ICD-9-D-29042, ICD-9-D-29043, ICD-9-D-29410, ICD-9-D-29411, ICD-9-D-29420, ICD-9-D-29421, ICD-9-D-33119, ICD-9-D-33182	ICD-10-D-F0150, ICD-10-D-F0151, ICD-10-D-F0280, ICD-10-D-F0281, ICD-10-D-F0390, ICD-10-D-F0391, ICD-10-D-G3109, ICD-10-D-G3183
Obesity	ICD-9-D-2780, ICD-9-D-27800, ICD-9-D-27801, ICD-9-D-27802, ICD-9-D-27803	ICD-10-D-E660:ICD-10-D-E669
Osteoarthritis	ICD-9-D-71500:ICD-9-D-71599	ICD-10-D-M1911:ICD-10-D-M1993
Parkinson’s disease	ICD-9-D-332, ICD-9-D-3320	ICD-10-D-G20, ICD-10-D-G214
Pulmonary heart disease	ICD-9-D-4150:ICD-9-D-41799	ICD-10-D-I26:ICD-10-D-I279
Rheumatoid arthritis	ICD-9-D-7140, ICD-9-D-7142	ICD-10-D-M0520:ICD-10-D-M061
Stroke	ICD-9-D-430, ICD-9-D-431, ICD-9-D-432, ICD-9-D-4320, ICD-9-D-4321, ICD-9-D-4329, ICD-9-D-43300, ICD-9-D-4331, ICD-9-D-43310, ICD-9-D-43311, ICD-9-D-43320, ICD-9-D-43321, ICD-9-D-43330, ICD-9-D-43331, ICD-9-D-43381, ICD-9-D-43390, ICD-9-D-43391, ICD-9-D-43400, ICD-9-D-43401, ICD-9-D-43410, ICD-9-D-43411, ICD-9-D-43490, ICD-9-D-43491	ICD-10-D-I6300, ICD-10-D-I63011, ICD-10-D-I63012, ICD-10-D-I63031, ICD-10-D-I63032, ICD-10-D-I6310, ICD-10-D-I63139, ICD-10-D-I63232, ICD-10-D-I63233, ICD-10-D-I63239, ICD-10-D-I63312, ICD-10-D-I6339, ICD-10-D-I6340, ICD-10-D-I63411, ICD-10-D-I63432, ICD-10-D-I6350, ICD-10-D-I63512, ICD-10-D-I63519, ICD-10-D-I63529, ICD-10-D-I6359, ICD-10-D-I638, ICD-10-D-I639
Tobacco use	ICD-9-D-3051, ICD-9-D-98984, ICD-9-D-V1582	ICD-10-D-F17220, ICD-10-D-F17221, ICD-10-D-F17223, ICD-10-D-F17228, ICD-10-D-F17229, ICD-10-D-F17290, ICD-10-D-F17291, ICD-10-D-F17293, ICD-10-D-F17298, ICD-10-D-F17299, ICD-10-D-Z720
Vascular dementia	ICD-9-D-29040, ICD-9-D-29041, ICD-9-D-29042, ICD-9-D-29043	ICD-10-D-F0150, ICD-10-D-F0151

*ICD, International Classification of Diseases.*

**TABLE 3:** Drug codes.

Insulin	GENERIC_DRUG-HUM_INSULIN_NPH/REG_INSULIN_HM, GENERIC_DRUG-INFUSION_SET-INSULIN_PUMP_BODY, GENERIC_DRUG-INFUSION_SET_FOR_INSULIN_PUMP, GENERIC_DRUG-INSULIN_ADMIN._SUPPLIES, GENERIC_DRUG-INSULIN_ASPART, GENERIC_DRUG-INSULIN_ASPART_(NIACINAMIDE), GENERIC_DRUG-INSULIN_ASPART_PROT/INSULN_ASP, GENERIC_DRUG-INSULIN_ASPART_PROTAM_&_ASPART, GENERIC_DRUG-INSULIN_DEGLUDEC, GENERIC_DRUG-INSULIN_DEGLUDEC/LIRAGLUTIDE, GENERIC_DRUG-INSULIN_DETEMIR, GENERIC_DRUG-INSULIN_GLARGINE/LIXISENATIDE, GENERIC_DRUG-INSULIN_GLARGINE_HUM.REC.ANLOG, GENERIC_DRUG-INSULIN_GLULISINE, GENERIC_DRUG-INSULIN_ISOPHANE_NPH_BF-PK, GENERIC_DRUG-INSULIN_LISPRO, GENERIC_DRUG-INSULIN_LISPRO_PROTAMIN/LISPRO, GENERIC_DRUG-INSULIN_NPH_HUM/REG_INSULIN_HM, GENERIC_DRUG-INSULIN_NPH_HUMAN_ISOPHANE, GENERIC_DRUG-INSULIN_NPL/INSULIN_LISPRO, GENERIC_DRUG-INSULIN_PUMP/INFUS._SET/METER, GENERIC_DRUG-INSULIN_PUMP_CARTRIDGE, GENERIC_DRUG-INSULIN_PUMP_CONTROLLER, GENERIC_DRUG-INSULIN_PUMP_SYRINGE__1.8_ML, GENERIC_DRUG-INSULIN_PUMP_SYRINGE__3_ML, GENERIC_DRUG-INSULIN_REGULAR_BEEF-PORK, GENERIC_DRUG-INSULIN_REGULAR__HUMAN, GENERIC_DRUG-INSULIN_ULTRALENTE, GENERIC_DRUG-INSULIN_ZINC_BEEF-PORK, GENERIC_DRUG-INSULN_ASP_PRT/INSULIN_ASPART, GENERIC_DRUG-NEEDLELESS_ACCESS._DEV_INSULIN, GENERIC_DRUG-NEEDLES__INSULIN_DISP.__SAFETY, GENERIC_DRUG-NEEDLES__INSULIN_DISPOSABLE, GENERIC_DRUG-NPH__HUMAN_INSULIN_ISOPHANE, GENERIC_DRUG-SUB-Q_INSULIN_DEVICE__20_UNIT, GENERIC_DRUG-SUB-Q_INSULIN_DEVICE__30_UNIT, GENERIC_DRUG-SUB-Q_INSULIN_DEVICE__40_UNIT, GENERIC_DRUG-SUBCUTANEOUS_INSULIN_PUMP, GENERIC_DRUG-SUBQ_INSULIN_PUMP_GLUC.MON.SYS, GENERIC_DRUG-SYRINGE-NEEDLE_INSULIN_0.5_ML, GENERIC_DRUG-SYRINGE_&_NEEDLE_INSULIN_1_ML, GENERIC_DRUG-SYRINGE_AND_NEEDLE_INSULIN_1ML, GENERIC_DRUG-SYRINGE_INSULIN_NEEDLESS_1_ML, GENERIC_DRUG-SYRINGE_W-NDL__DISP.__INSULIN, GENERIC_DRUG-SYRINGE_WITH_NEEDLE__INSULIN, GENERIC_DRUG-SYR_NDL_INSULIN_1ML-SHARPS_BIN
Metformin	GENERIC_DRUG-METFORMIN_HCL
Glitazones	GENERIC_DRUG-PIOGLITAZONE_HCL, GENERIC_DRUG-ROSIGLITAZONE_MALEATE
Sulfonylureas	GENERIC_DRUG-GLYBURIDE, GENERIC_DRUG-GLYBURIDE_MICRONIZED, GENERIC_DRUG-GLIMEPIRIDE, GENERIC_DRUG-GLIPIZIDE, GENERIC_DRUG-TOLAZAMIDE, GENERIC_DRUG-TOLBUTAMIDE, GENERIC_DRUG-CHLORPROPAMIDE
GLP1 Agonists	GENERIC_DRUG-EXENATIDE, GENERIC_DRUG-EXENATIDE_MICROSPHERES, GENERIC_DRUG-LIXISENATIDE, GENERIC_DRUG-ALBIGLUTIDE, GENERIC_DRUG-DULAGLUTIDE, GENERIC_DRUG-LIRAGLUTIDE, GENERIC_DRUG-SEMAGLUTIDE, GENERIC_DRUG-TEDUGLUTIDE
DPP4 inhibitors	GENERIC_DRUG-ALOGLIPTIN_BENZOATE, GENERIC_DRUG-LINAGLIPTIN, GENERIC_DRUG-SAXAGLIPTIN_HCL, GENERIC_DRUG-SITAGLIPTIN_PHOSPHATE
SGLT2 inhibitors	GENERIC_DRUG-CANAGLIFLOZIN, GENERIC_DRUG-DAPAGLIFLOZIN_PROPANEDIOL, GENERIC_DRUG-EMPAGLIFLOZIN, GENERIC_DRUG-ERTUGLIFLOZIN_PIDOLATE
Glinides	GENERIC_DRUG-NATEGLINIDE, GENERIC_DRUG-REPAGLINIDE
Metformin and sulfonylureas	GENERIC_DRUG-GLIPIZIDE/METFORMIN_HCL, GENERIC_DRUG-GLYBURIDE/METFORMIN_HCL, GENERIC_DRUG-GLYBURIDE__MICRO/METFORMIN_HCL
Metformin and glitazones	GENERIC_DRUG-PIOGLITAZONE_HCL/METFORMIN_HCL, GENERIC_DRUG-ROSIGLITAZONE/METFORMIN_HCL
Glitazones and sulfonylureas	GENERIC_DRUG-PIOGLITAZONE_HCL/GLIMEPIRIDE, GENERIC_DRUG-ROSIGLITAZONE/GLIMEPIRIDE

*DPP4, dipeptidyl-peptidase 4; GLP1, Glucagon-like Peptide 1; SGLT2, Sodium-glucose co-transporter 2.*

### Statistical Analysis

Statistical analyses were conducted between January 1st and February 28th, 2021. Patient demographic statistics and incidence statistics were analyzed using unpaired two-tailed *t*-tests or χ2 tests, as appropriate, to test the significance of the differences between continuous and categorical variables. In all analyses, a two-sided *P* < 0.05 was considered statistically significant.

A propensity score-matched population was generated by using a logistic regression to identify confounding factors for therapeutic treatment of T2D as outcome between the treatment and control groups as previously reported (Branigan et al., 2020; Torrandell-Haro et al., 2020). In brief, the resulting factors included age, region, gender, Charlson Comorbidity Index (CCI) rank as well as variable comorbidities including Asthma, Chronic Obstructive Pulmonary Disease (COPD), Chronic Kidney Disease, Congestive Heart Failure, Coronary Artery Disease, Hypertension, Obesity, Pulmonary Heart Disease, Ischemic Heart Disease, Tobacco Use, Osteoarthritis, Rheumatoid Arthritis, Stroke and Tobacco Use (Tables 2, 4). These factors were then used to match patients in the treatment group to patients in the control group to minimize confounding variables in the patient populations. The matching was assessed by standardized mean difference with percentage balance improvement (Table 5). Kaplan–Meier survival curves for AD-Free Survival were created using the propensity score-matched population in the Bellwether-PearlDiver interface.

**TABLE 4:** Baseline characteristics for unadjusted and propensity score-matched patients with or without exposure to anti-hyperglycemic medication.

	Unadjusted cohort					Propensity score-matched cohort*				
		
	Without exposure to A-HgM		With exposure to A-HgM			Without exposure to A-HgM		With exposure to A-HgM		
					
	*n*	%	*n*	%		*N*	%	*n*	%	
*Number of patients*	*638,625*		*1,176,407*		***p-value***	*638,625*		*638,625*		***p-value***
**Age**										
*45–49*	56,143	*8.79%*	122,003	*10.37%*	<*0.001*	56,143	*8.79%*	95,436	*14.94%*	<*0.001*
*50–54*	75,565	*11.83%*	166,337	*14.14%*		75,565	*11.83%*	118,983	*18.63%*	
*55–59*	87,529	*13.71%*	192,901	*16.40%*		87,529	*13.71%*	122,695	*19.21%*	
*60–64*	91,189	*14.28%*	193,543	*16.45%*		91,189	*14.28%*	107,415	*16.82%*	
*65–69*	93,519	*14.64%*	183,704	*15.62%*		93,519	*14.64%*	88,463	*13.85%*	
*70–74*	153,805	*24.08%*	237,322	*20.17%*		153,805	*24.08%*	93,780	*14.68%*	
*75–79*	80,875	*12.66%*	80,597	*6.85%*		80,875	*12.66%*	11,853	*1.86%*	
**Gender**										
*Female*	355,008	*55.59%*	623,613	*53.01%*	<*0.001*	355,008	*55.59%*	316,286	*49.53%*	<*0.001*
*Male*	283,617	*44.41%*	552,794	*46.99%*		283,617	*44.41%*	322,339	*50.47%*	
**Region**										
*Midwest*	115,866	*18.14%*	261,892	*22.26%*	<*0.001*	115,866	*18.14%*	177,665	*27.82%*	<*0.001*
*Northeast*	197,689	*30.96%*	228,000	*19.38%*		197,689	*30.96%*	33,773	*5.29%*	
*South*	242,852	*38.03%*	509,074	*43.27%*		242,852	*38.03%*	314,871	*49.30%*	
*West*	81,229	*12.72%*	175,411	*14.91%*		81,229	*12.72%*	111,137	*17.40%*	
*Unknown*	989	*0.15%*	2,030	*0.17%*		989	*0.15%*	1,179	*0.18%*	
**Comorbidities**										
*Asthma*	3,573	*0.56%*	74,503	*6.33%*	<*0.001*	3,573	*0.56%*	1,389	*0.22%*	<*0.001*
*COPD*	7,058	*1.11%*	44,892	*3.82%*	<*0.001*	7,058	*1.11%*	2,387	*0.37%*	<*0.001*
*Chronic kidney disease*	9,944	*1.56%*	163,445	*13.89%*	<*0.001*	9,944	*1.56%*	4,382	*0.69%*	<*0.001*
*Congestive heart failure*	11,956	*1.87%*	93,633	*7.96%*	<*0.001*	11,956	*1.87%*	4,966	*0.78%*	<*0.001*
*Coronary artery disease*	23,874	*3.74%*	236,139	*20.07%*	<*0.001*	23,874	*3.74%*	15,499	*2.43%*	<*0.001*
*Hypertension*	53,293	*8.34%*	542,014	*46.07%*	<*0.001*	53,293	*8.34%*	51,959	*8.14%*	<*0.001*
*Ischemic heart disease*	23,484	*3.68%*	189,927	*16.14%*	<*0.001*	23,484	*3.68%*	15,360	*2.41%*	<*0.001*
*Obesity*	18,079	*2.83%*	264,663	*22.50%*	<*0.001*	18,079	*2.83%*	18,191	*2.85%*	*0.55*
*Osteoarthritis*	26,365	*4.13%*	214,256	*18.21%*	<*0.001*	26,365	*4.13%*	14,140	*2.21%*	<*0.001*
*Pulmonary heart disease*	5,195	*0.81%*	53,546	*4.55%*	<*0.001*	5,195	*0.81%*	1,797	*0.28%*	<*0.001*
*Rheumatoid arthritis*	3,058	*0.48%*	20,362	*1.73%*	<*0.001*	3,058	*0.48%*	501	*0.08%*	<*0.001*
*Stroke*	12,676	*1.98%*	106,232	*9.03%*	<*0.001*	12,676	*1.98%*	4,450	*0.70%*	<*0.001*
*Tobacco use*	14,358	*2.25%*	119,678	*10.17%*	<*0.001*	14,358	*2.25%*	6,770	*1.06%*	<*0.001*
**CCI**										
*0–4*	586,371	*91.82%*	1,131,647	*96.20%*	<*0.001*	586,371	*91.82%*	635,303	*99.48%*	<*0.001*
*5–10*	48,527	*7.60%*	42,418	*3.61%*		48,527	*7.60%*	3,322	*0.52%*	
*11*+	3,727	*0.58%*	2,342	*0.20%*		3,727	*0.58%*	-	*0.00%*	

*A-HgM, anti-hyperglycemic medication; COPD, chronic obstructive pulmonary disease; CCI, Charlson comorbidity index.*

*Adjusted for age, sex, region, comorbidities, and Charlson comorbidity index (CCI).

**TABLE 5:** Summary of balance.

	All data			Matched data			% of balance improvement
			
	Means treated	Means control	Std. mean diff.	Means treated	Means control	Std. mean diff.	Std. mean diff.
Distance	0.672	0.604	0.132	0.754	0.604	0.132	–120.973
Age	62.036	63.877	9.382	59.351	63.877	9.382	–145.916
Gender F	0.530	0.556	0.497	0.495	0.556	0.497	–135.062
Gender M	0.470	0.444	0.497	0.505	0.444	0.497	–135.062
CCI	1.664	2.080	1.837	1.309	2.080	1.837	–85.384
regionNE	0.194	0.310	0.462	0.053	0.310	0.462	–121.757
regionSO	0.433	0.380	0.485	0.493	0.380	0.485	–114.955
regionUNKNOWN	0.002	0.002	0.039	0.002	0.002	0.039	–68.131
regionWE	0.149	0.127	0.333	0.174	0.127	0.333	–113.709
year2011	0.225	0.198	0.398	0.260	0.198	0.398	–131.551
year2012	0.166	0.160	0.367	0.174	0.160	0.367	–144.427
year2013	0.134	0.140	0.347	0.122	0.140	0.347	–168.772
year2014	0.166	0.173	0.378	0.157	0.173	0.378	–148.497
year2015	0.117	0.150	0.357	0.075	0.150	0.357	–130.485
year2016	0.076	0.098	0.297	0.046	0.098	0.297	–139.040
COMORB.Asthma	0.107	0.114	0.317	0.103	0.114	0.317	–67.179
COMORB.COPD	0.286	0.341	0.474	0.223	0.341	0.474	–116.371
COMORB.ChronicKidneyDisease	0.249	0.218	0.413	0.301	0.218	0.413	–173.375
COMORB.CongestiveHeartFailure	0.146	0.162	0.368	0.134	0.162	0.368	–77.990
COMORB.CoronaryArteryDisease	0.375	0.393	0.489	0.363	0.393	0.489	–66.575
COMORB.Hypertension	0.908	0.882	0.323	0.952	0.882	0.323	–161.252
COMORB.IschemicHeartDisease	0.313	0.344	0.475	0.282	0.344	0.475	–99.799
COMORB.Obesity	0.411	0.329	0.470	0.533	0.329	0.470	–149.535
COMORB.Osteoarthritis	0.369	0.445	0.497	0.272	0.445	0.497	–128.806
COMORB.PulmonaryHeartDisease	0.087	0.098	0.297	0.077	0.098	0.297	–86.365
COMORB.RheumatoidArthritis	0.037	0.059	0.236	0.015	0.059	0.236	–96.023
COMORB.TobaccoUse	0.210	0.229	0.420	0.192	0.229	0.420	–98.791
COMORB.Stroke	0.166	0.189	0.391	0.147	0.189	0.391	–81.683

*Std, standardized; Diff, difference; F, female; M, male; NE, northeast; SO, south; WE, west; COPD, chronic obstructive pulmonary disease.*

## Results

In the Mariner dataset available for analysis, 5,283,017 patients were identified with a diagnosis of Type 2 Diabetes (Figure 1). The exclusion and enrollment criteria for the study was met by 1,815,032 patients (Figure 1). After propensity score matching for age, gender, and comorbidities as previously described, 638,625 patients (mean age [SD]: 61.85 [6.2] years) were not exposed to A-HgM and controlled their diabetes through lifestyle whereas 638,625 patients (57.37 [5.6] years) were exposed to at least one therapeutic option (Figure 1). Mean (SD) follow-up was 6.2 (1.8) years.

In the unadjusted cohort, patients that did not receive medication for the treatment of T2D were older (51.38% vs. 43.18% patients 65 and older) and had lower comorbidity incidence at baseline (3.6 [Rheumatoid arthritis] to 11.3 [asthma] fold reduction when compared to drug exposed group) (Table 4). Cardiovascular disease (approximately 4–5 fold reduction), chronic kidney disease (8.9 fold reduction) and obesity (7.9 fold reduction), all risk factors that affect the incidence of dementia, were reduced in the population with diabetes controlled through lifestyle interventions (Table 4). In contrast, after adjusting for age, gender, and comorbidities, those patients who were not exposed to A-HgM had a slightly higher incidence of comorbidities than those who were treated with A-HgM (Table 4). To address the severity of T2D, the number of A-HgM drugs in the treated group was determined: 342,783 (56.68%) of patients were treated with 2 or less A-HgM drugs, 273,574 (42.84%) were exposed to 3 drugs, and 22,268 (3.49%) were exposed to 4.

**FIGURE 2:**
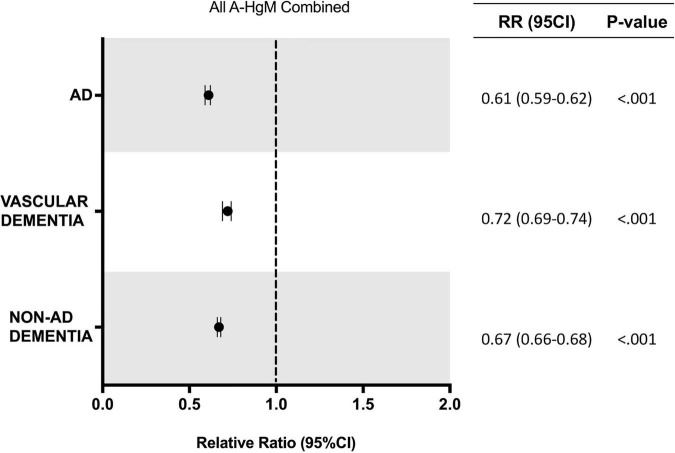
Relative risk of developing AD and ADRD for T2D patients with exposure to anti-hyperglycemic medication. AD, Alzheimer’s disease; RR, relative risk; CI, confidence interval.

**FIGURE 3:**
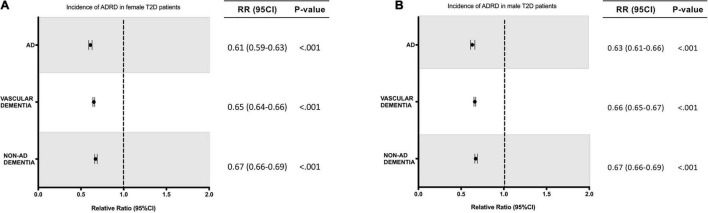
Relative risk of developing AD or ADRD for T2D women and men with exposure to anti-hyperglycemic medication. **(A)** Relative risk for T2D female patients. **(B)** Relative risk for T2D male patients. ADRD, Alzheimer’s disease related dementias; AD, Alzheimer’s disease; RR, relative risk; CI, confidence interval.

In the population receiving A-HgM, there was an overall decreased risk of a newly diagnosed AD (2.18% vs. 2.68%, RR: 0.81; 95% CI: 0.80–0.83, *p* < 0.001), VasD (1.14% vs. 1.28%, RR: 0.89; 95% CI: 0.87–0.91, *p* < 0.001) or non-AD dementia (4.62% vs. 5.45%, RR: 0.85; 95% CI: 0.84–0.86, *p* < 0.001) (Table 6). After propensity score matching, exposure to A-HgM agents was associated with a decreased incidence of AD (1.63% vs. 2.68%, RR: 0.61; 95% CI: 0.59–0.62, *p* < 0.001), VasD (0.91% vs. 1.28%, RR: 0.72; 95% CI: 0.69–0.74, *p* < 0.001) and non-AD dementia (3.64% vs. 5.45%, RR: 0.67; 95% CI: 0.66–0.68, *p* < 0.001) compared to control patients (lifestyle controlled/non-A-HgM exposure) (Table 6 and Figure 2). The number of patients needed to treat to reduce risk of AD, VasD, and non-AD dementia was 95.32, 274.9 and 55.26 respectively in the adjusted population (Table 6). Additional analyses indicated no significant sex differences in AD and ADRD risk reduction profiles between males and females when evaluated separately (Figure 3).

**TABLE 6:** Incidence and relative risk of patients taking anti-hyperglycemic medication to develop AD and ADRD.

	AD	Vascular dementia	Non-AD dementia
**Unadjusted cohort**			
Patients not receiving A-HgM	*17,086*	*8,161*	*34,694*
%	2.68%	1.28%	5.45%
Patients receiving A-HgM	*25,711*	*13,450*	*54,498*
%	2.18%	1.14%	4.62%
Relative Risk	*0.81*	*0.89*	*0.85*
95%CI	0.80–0.83	0.87–0.91	0.84–0.86
NNT	198.8	709.1	120.9
*p-value*	<*0.001*	<*0.001*	<*0.001*
Propensity score-matched cohort*			
Patients not receiving A-HgM	*17,134*	*8,161*	*34,790*
%	2.68%	1.28%	5.45%
Patients receiving A-HgM	*10,434*	*5,838*	*23,234*
%	1.63%	0.91%	3.64%
Relative risk	*0.61*	*0.72*	*0.67*
95%CI	0.59–0.62	0.69–0.74	0.66–0.68
NNT	95.32	274.9	55.26
*p-value*	<*0.001*	<*0.001*	<*0.001*

*ADRD, Alzheimer’s disease related dementias; AD, Alzheimer’s disease; A-HgM, anti-hyperglycemic medication; CI, confidence interval; NNT, number needed to treat.*

*Adjusted for age, sex, region, comorbidities, and Charlson comorbidity index (CCI).

**FIGURE 4:**
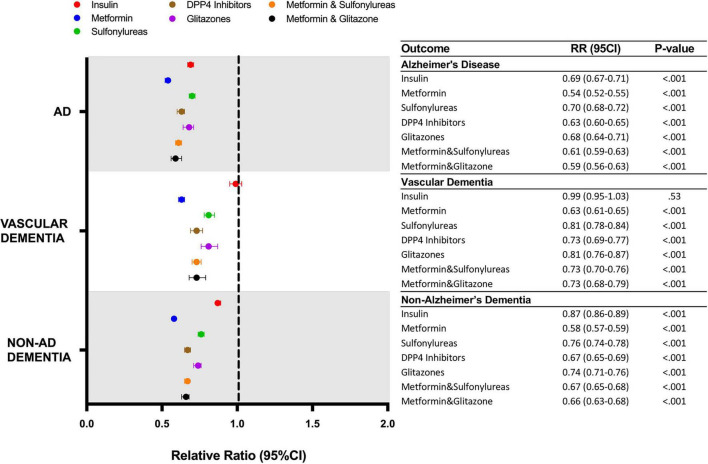
Relative risk of developing AD or ADRD for T2D patients receiving different classes of anti-hyperglycemic medication. AD, Alzheimer’s disease; DPP4, dipeptidyl-peptidase 4; RR, relative risk; CI, confidence interval.

Within the propensity score matched population, the association of individual classes of drugs on AD, VasD and non-AD dementia outcomes was evaluated (Figure 4). Metformin was associated with the greatest reduction in risk of AD (1.44% vs. 2.68%, RR: 0.54; 95% CI: 0.52-0.55, *p* < 0.001) VasD (0.80% vs. 1.28%, RR: 0.63; 95% CI: 0.61–0.65, *p* < 0.001) and non-AD dementia (3.16% vs. 5.45%, RR: 0.58; 95% CI: 0.57–0.59, *p* < 0.001). The association between metformin and reduced ADRD risk was significantly greater than all other drug categories and showed non-overlapping 95% confidence intervals within a disease outcome (Figure 4). For incidence of non-AD dementia, insulin (4.76% vs. 5.45%, RR: 0.87; CI: 0.86–0.89, *p*-value < 0.001) use was associated with a significantly lower risk reduction than all other drug categories. For VasD, insulin was not associated with a change in risk (1.26%, vs. 1.28%, RR: 0.99; 95% CI: 0-95–1.03, *p* = 0.53) compared to control. For a new diagnosis of AD, insulin showed a smaller relative risk reduction than metformin, DDP4 inhibitors and metformin combined with sulfonylureas or glitazones.

Differences in the baseline characteristics of patients exposed to insulin and those exposed to metformin were determined (Table 7). The population exposed to insulin were slightly older and had a higher incidence of comorbidities. Specifically, they had a higher incidence of chronic kidney disease, congestive heart failure, coronary artery disease, pulmonary heart disease, and stroke (Table 7). Based on these differences, determination of baseline comorbidities of patients that developed AD while using insulin or metformin was investigated (Table 8). Patients who developed AD in both insulin and metformin exposed groups were older and predominantly female compared to patients who did not develop AD (Table 8). Patients who developed AD had an overall higher incidence of comorbidities but surprisingly, had a significantly lower incidence of obesity in both metformin and insulin populations compared to patients who did not develop AD. When comparing patients who developed AD with exposure to metformin versus exposure to insulin, there were significant differences in age and comorbidity rates. Patients who developed AD after metformin exposure were older than those who developed AD after insulin exposure. However, patients who developed AD after being exposed to insulin had a higher incidence of COPD, chronic kidney disease, chronic heart failure, coronary artery disease, obesity and stroke but had less incidence of hypertension (Table 8).

**TABLE 7:** Patient demographics and comorbidities in patients treated with insulin in comparison with patients treated with metformin.

	With insulin exposure		With metformin exposure		
		
	*n*	%	*n*	%	
	
*Number of patients*	*250,811*		*523,758*		*p-value*
**Age**					
*45–49*	38,086	*15.19%*	82,605	*15.77%*	<*0.001*
*50–54*	47,153	*18.80%*	102,320	*19.54%*	
*55–59*	48,687	*19.41%*	103,949	*19.85%*	
*60–64*	42,640	*17.00%*	88,681	*16.93%*	
*65–69*	34,376	*13.71%*	71,545	*13.66%*	
*70–74*	35,724	*14.24%*	66,322	*12.66%*	
*75–79*	4,145	*1.65%*	8,336	*1.59%*	
**Gender**					
*Female*	123,517	*49.25%*	261,627	*49.95%*	<*0.001*
*Male*	127,294	*50.75%*	262,131	*50.05%*	
**Region**					
*Midwest*	70,402	*28.07%*	146,773	*28.02%*	*0.78*
*Northeast*	13,994	*5.58%*	27,862	*5.32%*	
*South*	120,968	*48.23%*	256,291	*48.93%*	
*West*	44,951	*17.92%*	91,953	*17.56%*	
*Unknown*	496	*0.20%*	879	*0.17%*	
**Comorbidities**					
*Asthma*	14,626	*5.83%*	26,366	*5.03%*	<*0.001*
*COPD*	8,161	*3.25%*	11,665	*2.23%*	<*0.001*
*Chronic kidney disease*	50,850	*20.27%*	47,393	*9.05%*	<*0.001*
*Congestive heart failure*	23,710	*9.45%*	23,922	*4.57%*	<*0.001*
*Coronary artery disease*	52,888	*21.09%*	78,621	*15.01%*	<*0.001*
*Hypertension*	103,381	*41.22%*	218,014	*41.62%*	< *0.001*
*Obesity*	63,732	*25.41%*	130,119	*24.84%*	< *0.001*
*Osteoarthritis*	31,027	*12.37%*	61,938	*11.83%*	<*0.001*
*Pulmonary heart disease*	12,404	*4.95%*	14,063	*2.69%*	<*0.001*
*Rheumatoid arthritis*	2,013	*0.80%*	3,278	*0.63%*	<*0.001*
*Stroke*	22,565	*9.00%*	30,919	*5.90%*	<*0.001*
*Tobacco use*	22,195	*8.85%*	43,652	*8.33%*	<*0.001*
**CCI**					
*0*	16,015	*6.39%*	39,012	*7.45%*	<*0.001*
*1*	156,081	*62.23%*	359,509	*68.64%*	
*2*	49,442	*19.71%*	89,187	*17.03%*	
*3*	20,329	*8.11%*	27,853	*5.32%*	
*4*	6,802	*2.71%*	6,594	*1.26%*	
*5–10*	2,142	*0.85%*	1,603	*0.31%*	
*11*+	–	*0.00%*	–	*0.00%*	

*AD, Alzheimer’s disease; COPD, chronic obstructive pulmonary disease; CCI, Charlson comorbidity index.*

**TABLE 8:** Patient demographics and comorbidities in patients who develop and who do not develop AD treated with insulin in comparison with patients treated with metformin.

	Develop AD after exposure to insulin		Do not develop AD after exposure to insulin			Develop AD after exposure to metformin		Do not develop AD after exposure to metformin			Develop AD insulin vs. metformin	Do not develop AD insulin vs. metformin
					
	*n*	%	*n*	%		*n*	%	*n*	%			

*Number of patients*	*4,557*		*246,169*		***p-value***	*7,363*		*516,230*		***p-value***	***p-value***	***p-value***
**Age**												
*45–49*	49	*1.08%*	38,032	*15.45%*	<*0.001*	78	*1.06%*	82,524	*15.99%*	<*0.001*	<*0.01*	<*0.001*
*50–54*	124	*2.72%*	47,027	*19.10%*		182	*2.47%*	102,132	*19.78%*			
*55–59*	252	*5.53%*	48,430	*19.67%*		358	*4.86%*	103,578	*20.06%*			
*60–64*	485	*10.64%*	42,151	*17.12%*		712	*9.67%*	87,950	*17.04%*			
*65–69*	857	*18.81%*	33,508	*13.61%*		1,343	*18.24%*	70,178	*13.59%*			
*70–74*	2,486	*54.55%*	33,195	*13.48%*		4,183	*56.81%*	62,064	*12.02%*			
*75–79*	304	*6.67%*	3,826	*1.55%*		507	*6.89%*	7,804	*1.51%*			
**Gender**												
*Female*	2,532	*55.56%*	120,933	*49.13%*	<*0.001*	4,108	*55.79%*	257,430	*49.87%*	<*0.001*	*0.81*	<*0.001*
*Male*	2,025	*44.44%*	125,236	*50.87%*		3,255	*44.21%*	258,800	*50.13%*			
**Region**												
*Midwest*	1,350	*29.62%*	69,034	*28.04%*	*0.04*	2,204	*29.93%*	144,531	*28.00%*	*0.03*	*0.86*	*0.74*
*Northeast*	153	*3.36%*	13,840	*5.62%*		218	*2.96%*	27,642	*5.35%*			
*South*	2,338	*51.31%*	118,585	*48.17%*		3,733	*50.70%*	252,467	*48.91%*			
*West*	701	*15.38%*	44,230	*17.97%*		1,192	*16.19%*	90,728	*17.58%*			
*Unknown*	15	*0.33%*	480	*0.19%*		16	*0.22%*	862	*0.17%*			
**Comorbidities**												
*Asthma*	192	*4.21%*	14,425	*5.86%*	<*0.001*	258	*3.50%*	26,128	*5.06%*	<*0.001*	*0.05*	<*0.001*
*COPD*	191	*4.19%*	7,989	*3.25%*	<*0.001*	218	*2.96%*	11,460	*2.22%*	<*0.001*	<*0.001*	<*0.001*
*Chronic kidney disease*	1,355	*29.73%*	49,588	*20.14%*	<*0.001*	1,619	*21.99%*	46,229	*8.96%*	<*0.001*	<*0.001*	<*0.001*
*Chronic heart failure*	665	*14.59%*	23,066	*9.37%*	<*0.001*	688	*9.34%*	23,331	*4.52%*	<*0.001*	<*0.001*	<*0.001*
*Coronary artery disease*	1,309	*28.73%*	51,596	*20.96%*	<*0.001*	1,838	*24.96%*	76,964	*14.91%*	<*0.001*	<*0.001*	<*0.001*
*Ischemic heart disease*	1,075	*23.59%*	39,377	*16.00%*	<*0.001*	1,481	*20.11%*	57,966	*11.23%*	<*0.001*	<*0.001*	<*0.001*
*Hypertension*	2,163	*47.47%*	101,217	*41.12%*	<*0.001*	3,669	*49.83%*	214,404	*41.53%*	<*0.001*	*0.01*	<*0.001*
*Obesity*	837	*18.37%*	62,871	*25.54%*	<*0.001*	1,220	*16.57%*	128,907	*24.97%*	<*0.001*	*0.01*	<*0.001*
*Osteoarthritis*	784	*17.20%*	30,246	*12.29%*	<*0.001*	1,254	*17.03%*	60,766	*11.77%*	<*0.001*	*0.81*	<*0.001*
*Pulmonary heart disease*	241	*5.29%*	12,139	*4.93%*	*0.27*	345	*4.69%*	13,792	*2.67%*	<*0.001*	*0.14*	<*0.001*
*Rheumatoid arthritis*	33	*0.72%*	1,973	*0.80%*	*0.56*	54	*0.73%*	3,223	*0.62%*	*0.24*	*0.95*	<*0.001*
*Stroke*	844	*18.52%*	21,787	*8.85%*	<*0.001*	1,208	*16.41%*	29,895	*5.79%*	<*0.001*	<*0.01*	<*0.001*
*Tobacco use*	356	*7.81%*	21,844	*8.87%*	<*0.01*	551	*7.48%*	43,107	*8.35%*	<*0.001*	*0.51*	<*0.001*
**CCI**												
*0*	181	*3.97%*	15,823	*6.43%*	*0.01*	230	*3.12%*	38,774	*7.51%*	<*0.001*	<*0.001*	<*0.001*
*1*	2,849	*62.52%*	153,188	*62.23%*		5,132	*69.70%*	354,271	*68.63%*			
*2*	1,004	*22.03%*	48,422	*19.67%*		1,448	*19.67%*	87,708	*16.99%*			
*3*	392	*8.60%*	19,928	*8.10%*		430	*5.84%*	27,407	*5.31%*			
*4*	109	*2.39%*	6,691	*2.72%*		104	*1.41%*	6,490	*1.26%*			
*5–10*	22	*0.48%*	2,117	*0.86%*		19	*0.26%*	1,580	*0.31%*			
*11*+	–	*0.00%*	–	*0.00%*		–	*0.00%*	–	*0.00%*			

*AD, Alzheimer’s disease; COPD, chronic obstructive pulmonary disease; CCI, Charlson comorbidity index.*

**FIGURE 5:**
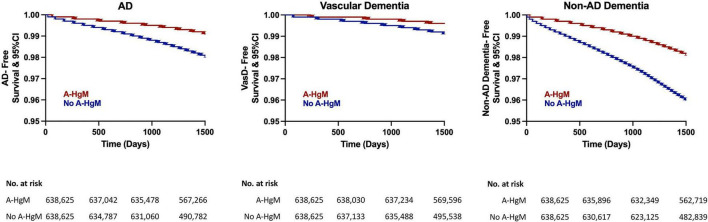
Kaplan–Meier survival curves for disease-free survival for AD and ADRD subtype (vascular dementia and non-AD dementia). AD, Alzheimer’s disease; A-HgM, anti-hyperglycemic medication; CI, confidence interval.

In Kaplan–Meier survival curves, depicting disease development over time, exposure to A-HgM was associated with a reduced conversion to AD and ADRD with the greatest risk reduction occurring in non-AD dementia outcomes followed by AD with A-HgM exposure showing the smallest risk reduction profile in VasD (Figure 5).

## Discussion

There is growing evidence that use of A-HgM medications has the potential to reduce AD and ADRD risk (Bohlken et al., 2018; Boccardi et al., 2019). Based on this body of evidence, clinical trials to test the effects of A-HgM in patients with AD were conducted (Geldmacher et al., 2011; Koenig et al., 2017; Craft et al., 2020). In a small trial in 20 non-diabetic patients with MCI or mild dementia due to AD, metformin improved executive function (Trails-B test) (Koenig et al., 2017) which has led to an ongoing Phase II/III trial assessing metformin in a comparable population (NCT01965756). DPP4 inhibitors also have been reported to protect against cognitive decline in diabetic patients (Rizzo et al., 2014). Intranasal insulin administered over 12 months to patients with MCI or mild dementia due to AD did not show cognitive or functional improvement (Craft et al., 2020). Inconsistent results have been observed in clinical studies testing the effects of pioglitazone in AD patients (Geldmacher et al., 2011; Sato et al., 2011). However, these studies were small and evaluated the effects of A-HgM therapeutics in patients, not all T2D, with memory decline. The results from this study involve the evaluation of A-HgM exposure for prevention of AD and ADRD, not treatment, in patients with a risk factor for AD, T2D.

Overall, results of analyses reported herein indicated a positive association of anti-hyperglycemic exposure in T2D patients with reduced risk of AD and AD related dementias (ADRD) outcomes. In our initial non-adjusted analysis, patients without therapeutic intervention for diabetes were older, but had reduced incidence of several comorbidities (pulmonary and cardiovascular disease, arthritis, and obesity) and reduced tobacco use compared to control. These observations are consistent with clinical practice in which patients that control their diabetes with lifestyle changes tend to be healthier overall (Colberg et al., 2016). As many of these comorbidities are risk factors for the development of AD and ADRD, propensity score matching of the populations was conducted to isolate the contribution of A-HgM exposure alone. The association between anti-hyperglycemic agents and reduced risk was observed in both the unadjusted and adjusted populations. After matching for covariates of interest (Table 4), the association for reduced risk with A-HgM exposure in the propensity score matched population was greater than that observed in the unadjusted population suggesting that the comorbidities of interest can impact the efficacy of these therapies to reduce risk of AD and ADRD (Table 6 and Figure 2).

Metformin was associated with the greatest risk reduction in AD, VasD and non-AD dementia outcomes whereas insulin was associated with a smaller reduction in risk and was not associated with any change in VasD risk. This is consistent with a recent publication (Weinstein et al., 2019) that analyzed data pooled from five longitudinal cohorts where insulin use was associated with increased risk of developing dementia and decline in global cognitive function which led the authors to hypothesize that the increase in cognitive decline could be due to increased incidence of hypoglycemia. Of interest, T2D patients who developed AD were older and predominantly female compared to patients who did not develop AD. This is consistent with AD epidemiological data where age and female sex are the primary risk factors for the development of AD. These finding warrant further studies to determine potential subsets of responders and non-responders to these therapeutics. Taken together, our results support the hypothesis that early and effective control of hyperglycemia has the potential to reduce risk for AD and ADRD in the setting of T2D. Anti-hyperglycemic therapies can affect specific pathways of action of glucose metabolism and thus result in differential risk profiles based on the systems of biology affected by each therapeutic.

### Limitations

This analysis has several limitations. First, as this study is a retrospective analysis of a claims database patients included may have obtained services outside of those included in this database for which we would not be able to address. Second, there could be factors, known and unknown, that even with propensity matching may not be adequately addressed. Thus, as with all observational studies, there are opportunities for bias in the interpretation of these data. We have attempted to address these through the use of propensity score matching and in our study design wherever possible. Third, lifestyle modifications, which are commonly used in the early stages of disease, could not be addressed in this study. Additionally, this dataset does not include information on glycemic index of the patients Fourth, this dataset does not contain data on ethnicity or race and therefore, those were not assessed in the analysis. Fifth, diagnosis of disease relied on the physician diagnosis and the ICD code assigned to each patient presentation. As the diagnosis of AD and ADRD is clinical, there may be overlap between AD and dementia codes given similar presentations despite different underlying pathophysiology. Furthermore, there could be biases in the prescribing trends for A-HgM that cannot be controlled in the model. Lastly, we did not address switching of A-HgM between therapeutic groups in our analysis.

## Conclusion

Analysis of specific classes of A-HgM therapeutics advance a precision medicine approach for the prevention of Alzheimer’s Disease and related dementias. Exposure to anti-hyperglycemic agents was associated with reduced incidence of AD and ADRD. Further, individual therapies within A-HgM exert different risk reduction profiles with metformin exerting the greatest risk reduction. As T2D affects more than 400 million persons world-wide and is a risk factor for AD and ADRD, targeted A-HgM therapeutics to reduce risk of AD and ADRD could substantially impact long-term neurological health outcomes.

## Data Availability Statement

The data analyzed in this study is subject to the following licenses/restrictions: Restrictions apply to the availability of some or all data generated or analyzed during this study to preserve patient confidentiality or because they were used under license. The corresponding author will on request detail the restrictions and any conditions under which access to some data may be provided. Requests to access these datasets should be directed to info@pearldiverinc.com.

## Author Contributions

GT-H, GB, KR, and RB: full access to all the data in the study and take responsibility for the integrity of the data and the accuracy of the data analysis. RB and KR: obtain funding, administrative, technical, material support, supervision, and guarantor. GT-H and GB: statistical analysis. All authors: concept and design, acquisition, analysis, interpretation of data, drafting of the manuscript, and critical revision of the manuscript for important intellectual content.

## Conflict of Interest

RB reported receiving grants from the Women’s Alzheimer’s Movement and the National Institute on Aging during the conduct of the study. KR reported receiving grants from the National Institute on Neurological Disease and Stroke during the conduct of the study. The remaining authors declare that the research was conducted in the absence of any commercial or financial relationships that could be construed as a potential conflict of interest.

## Publisher’s Note

All claims expressed in this article are solely those of the authors and do not necessarily represent those of their affiliated organizations, or those of the publisher, the editors and the reviewers. Any product that may be evaluated in this article, or claim that may be made by its manufacturer, is not guaranteed or endorsed by the publisher.
